# Metabolic Deficits in the Retina of a Familial Dysautonomia Mouse Model

**DOI:** 10.3390/metabo14080423

**Published:** 2024-07-31

**Authors:** Stephanann M. Costello, Anastasia Schultz, Donald Smith, Danielle Horan, Martha Chaverra, Brian Tripet, Lynn George, Brian Bothner, Frances Lefcort, Valérie Copié

**Affiliations:** 1 Department of Chemistry and Biochemistry, Montana State University—Bozeman, Bozeman, MT 59717, USA; scostello@mtech.edu (S.M.C.);; 2 Department of Microbiology and Cell Biology, Montana State University—Bozeman, Bozeman, MT 59717, USA; 3 Department of Biological and Physical Sciences, Montana State University—Billings, Billings, MT 59102, USA

**Keywords:** conditional knockout (CKO) FD mouse model, eye health, familial dysautonomia (FD), metabolomics, nuclear magnetic resonance, mass spectrometry, retinal immunohistology, retinal metabolism, retinal ganglion cells, dopaminergic amacrine cells, univariate and multivariate statistical analyses

## Abstract

Neurodegenerative retinal diseases such as glaucoma, diabetic retinopathy, Leber’s hereditary optic neuropathy (LHON), and dominant optic atrophy (DOA) are marked by progressive death of retinal ganglion cells (RGC). This decline is promoted by structural and functional mitochondrial deficits, including electron transport chain (ETC) impairments, increased oxidative stress, and reduced energy (ATP) production. These cellular mechanisms associated with progressive optic nerve atrophy have been similarly observed in familial dysautonomia (FD) patients, who experience gradual loss of visual acuity due to the degeneration of RGCs, which is thought to be caused by a breakdown of mitochondrial structures, and a disruption in ETC function. Retinal metabolism plays a crucial role in meeting the elevated energetic demands of this tissue, and recent characterizations of FD patients’ serum and stool metabolomes have indicated alterations in central metabolic processes and potential systemic deficits of taurine, a small molecule essential for retina and overall eye health. The present study sought to elucidate metabolic alterations that contribute to the progressive degeneration of RGCs observed in FD. Additionally, a critical subpopulation of retinal interneurons, the dopaminergic amacrine cells, mediate the integration and modulation of visual information in a time-dependent manner to RGCs. As these cells have been associated with RGC loss in the neurodegenerative disease Parkinson’s, which shares hallmarks with FD, a targeted analysis of the dopaminergic amacrine cells and their product, dopamine, was also undertaken. One dimensional (1D) proton (^1^H) nuclear magnetic resonance (NMR) spectroscopy, mass spectrometry, and retinal histology methods were employed to characterize retinae from the retina-specific *Elp1* conditional knockout (CKO) FD mouse model (*Pax6-Cre*; *Elp1^LoxP/LoxP^*). Metabolite alterations correlated temporally with progressive RGC degeneration and were associated with reduced mitochondrial function, alterations in ATP production through the Cahill and mini-Krebs cycles, and phospholipid metabolism. Dopaminergic amacrine cell populations were reduced at timepoints P30–P90, and dopamine levels were 25–35% lower in CKO retinae compared to control retinae at P60. Overall, this study has expanded upon our current understanding of retina pathology in FD. This knowledge may apply to other retinal diseases that share hallmark features with FD and may help guide new avenues for novel non-invasive therapeutics to mitigate the progressive optic neuropathy in FD.

## 1. Introduction

Familial dysautonomia (FD) is a rare autosomal recessive, developmental, and progressive neurodegenerative disease that impacts the central and peripheral nervous systems. The disease is caused by a single point mutation (c.2204 + 6T > C) in intron 20 of the elongator protein 1 (*ELP1*, formerly *IKBKAP*)-coding gene, resulting in a tissue-specific deficiency of ELP1 [1]. Neurons are the most impacted cell type in FD, producing little functional protein [1,2,3]. FD is classified as hereditary sensory and autonomic neuropathy (HSAN type III), and patients present with highly variable phenotypes marked by pain and temperature sensation deficits, cardiovascular instability, spinal curvature, gastrointestinal (GI) dyscoordination, gait ataxia, low body mass index, and progressive visual decline [2,3,4,5]. FD is highly debilitating with a 50% mortality rate by the age of 40 [1,2]. The loss of vision is one of the few phenotypes ubiquitous to all patients, and taken together with gait ataxia, visual decline becomes one of the most debilitating symptoms impacting FD patients’ quality of life [6]. The visual impairment developed by FD patients is due to progressive optic neuropathy and death of retinal ganglion cells (RGCs). In particular, the temporal optic nerve is considerably reduced, resulting in a central field of view deficits and color vision defects [6,7,8]. Clinical findings present with reduced papillomacular retinal nerve fiber layer (RNFL) thickness and, upon autopsy, reduced mitochondrial density was detected in the temporal portion of the optic nerve [6]. These findings have been corroborated in our FD mouse models that displayed mitochondrial dysfunction, elevated reactive oxygen species (ROS), and reduced ATP production [6,7,8,9,10] and are reminiscent of well-established optic neuropathies such as Leber’s heredity optic neuropathy (LHON) and Dominant optic atrophy (DOA) that are due to mitochondrial dysfunction.

In neurodegenerative retinal diseases such as glaucoma and diabetic retinopathy, RGCs are the primary cell type affected. However, studies have observed cell death in other retinal layers, such as the amacrine cells of the inner nuclear layer (INL) [11,12]. Amacrine cells are interneurons that are synaptically active in the inner plexiform layer (IPL), integrating and modulating information in a time-dependent manner to the RGCs [13,14]. There exists a variety of amacrine cell subtypes, each playing an integral role in signal transmission in the retina. One key subpopulation is the dopaminergic amacrine cells, which play a critical role in modulating light adaptation and color vision by affecting synaptic transmission [13,14,15].

In Parkinson’s disease (PD), a neurodegenerative disease characterized by the death of dopaminergic neurons in the Substantia nigra, many patients present with visual impairment similar to what is observed in FD, including decreased visual acuity, color blind defects, and difficulties performing more complex visual tasks [16]. It has also been reported that the number of dopaminergic amacrine cells is reduced in the PD retinae [17]. PD patients also display a reduction in the RNFL similar to the clinical findings of axonal loss in the papillomacular bundle found in LHON, DOA, and FD, and indicative of altered mitochondrial function [16,18,19]. Thus, we hypothesized that a similar phenomenon of dopaminergic amacrine cell atrophy may be occurring in FD. Thus, we hypothesized that a similar phenomenon of dopaminergic amacrine cell atrophy may be occurring in FD.

Collectively, these data indicate disruptions in cellular energetics in FD. To directly investigate the metabolism in FD, we recently characterized the metabolomes of FD patients’ stool and serum samples and compared them to those of healthy control relatives using 1D (1-dimensional) ^1^H (proton) nuclear magnetic resonance (NMR) metabolomics techniques. This work demonstrated that, in fact, the metabolome of FD patients differs significantly from their cohabitating healthy (heterozygous for the *ELP1* mutation) relatives and provided valuable insights into the link between alterations in central metabolic pathways, progressive neurodegeneration, and clinical phenotypes of FD patients [20,21].

The retina is a particularly energetically demanding tissue and utilizes specialized metabolic mechanisms to accommodate its energetic needs [22]. Given mitochondrial dysfunction and preferential loss of RGCs observed in FD patients and mouse models [6,7,8,9,10], in conjunction with energetic deficits revealed in the analysis of FD patients’ stool and serum metabolite profiles [20,21], we sought to characterize temporal changes in the retinal metabolome as a function of the progressive neurodegeneration observed in the *Pax6-Cre*; *Elp1^LoxP/LoxP^* FD mouse model. To this end, 1D ^1^H NMR metabolomics and targeted mass spectrometry analyses were undertaken, together with retinal histology, to characterize metabolic patterns of FD mouse retinae. Our overarching goal has been to examine how metabolic alterations correlate with RGC neurodegeneration over time, whether loss of RGCs alters metabolic homeostasis of the retina, and if amacrine cell populations are impacted in this FD mouse model.

Limited energy (ATP) production as a result of mitochondrial dysfunction is associated with retinal diseases [12,23,24,25]. The goal of the present work has been to generate new molecular knowledge about the energetics of the FD retina, how perturbations in retinal metabolism leads to pathology, and to identify metabolic pathways that could be targeted for treatment of the FD retina and potentially other optic neuropathies [26], such as LHON and DOA, which share the similar trait of mitochondrial dysfunction with FD.

## 2. Materials and Methods

### 2.1. Mice

Mouse studies were conducted in the American Association for Laboratory Animal Care (AALAC) accredited Animal Resource Center at Montana State University under the IACUC-approved protocol (MSU protocol no. 2020-15-51). Retina-specific *Elp1* conditional knockout mice (CKO), *Pax6-Cre*; *Elp1^LoxP/LoxP^*, were generated as described [10]. Littermates (*Elp1^LoxP/LoxP^*) were used as control mice (hereafter referred to as control). Four time points throughout disease progression were used for experimentation to track RGC death and monitor metabolic changes, as well as changes in other retinal neurons: 7 days (P7), 30 days (P30), 60 days (P60), and 90 days (P90). P7 and P60 were used to examine the retina metabolome by NMR. For P7, 14 retina pairs (i.e., 14 animals) were used. For P60, 17 retina pairs were used, which consisted of 9 CKOs (4 males and 5 females) and 8 controls (2 males and 6 females). P30 was used for MS experiments Run 1 and Run 2, which used 14 and 20 retina pairs, respectively. Out of the 14 animals for Run 1, 6 were CKOs (2 males and 4 females), and 8 were controls (3 males and 5 females). Out of the 20 animals for Run 2, 10 were CKOs (4 males and 6 females), and 8 were controls (7 males and 3 females). For retinal histology and dopaminergic amacrine (DA) cell analysis, the time points included P7, P30, and P90. Mice were housed under a 14 h light/10 h dark cycle with temperatures of 18–23 °C and 40–60% humidity, with food (irradiated PicoLab^®^ Rodent Diet 20, 5053 LabDiet^®^, Land O’Lakes, Inc., Arden Hills, MN, USA) and water given ad libitum.

### 2.2. Retina Sample Collection for NMR and MS Metabolite Extractions

P7 mice were humanely euthanized by carbon dioxide (CO_2_) exposure along with rapid decapitation in accordance with MSU IACUC-approved procedures (MSU protocol No. 2021-35-81). A small incision was then made to the eyelid, and the eyes were enucleated. Retinae were rapidly dissected while eyes were suspended in cold phosphate-buffered saline (PBS). The cornea was excised from the sclera, the lens was removed, and the retina was peeled away from the eyecup [9,10,27]. Retina pairs from each animal were collected and flash-frozen in a 95% ethanol dry ice bath. Thus, each sample consisted of both retinae from each mouse. Retinae from P30 and P60 mice were collected in the same manner. However, following euthanasia by CO_2_ exposure, the mice were cervically dislocated, and their eyes were enucleated. Retinae were stored at −80 °C until further processed for NMR or MS metabolite extractions.

### 2.3. Metabolite Extraction for ^1^H NMR Analysis

A perchloric acid-based metabolite extraction that was optimized to measure taurine levels and had been used for ^1^H NMR analysis of rabbit cornea [28] and rat retina [29] was adapted and modified for the extraction of polar metabolites from FD mouse retinae collected at P7 and P60. Retinae were homogenized with a micro-pestle immediately after retrieval from storage at −80 °C, while the tissue was still frozen. A volume of 500 µL of 7% perchloric acid was added to the homogenized tissue, and the samples were vortexed for ~1 min. Following homogenization, all samples were centrifuged at 4000× *g* for 12 min. The supernatant was transferred to a new 1.5 mL microcentrifuge tube, followed by the addition of 10 µL of 1M phenol red indicator stock solution in NMR buffer. The sample was neutralized with 10 M KOH stock solution based on visualization of pH indicator changes and formation of a white KClO_4_ precipitate. A faint yellow color was observed following the addition of 80 µL of 10 M KOH, indicating a sample pH of 7.0. The sample was then centrifuged (4000× *g* for 7 min) to remove the KClO_4_ precipitate, and the supernatant was transferred to a new 1.5 mL microcentrifuge tube. The resulting metabolite mixture was concentrated by drying overnight in a speed vacuum concentrator without heat, and then reconstituted in 600 µL of NMR buffer consisting of 0.25 mM sodium 2, 2-Dimethyl-2-silapentane-5-sulfonic (DSS) in 90% H_2_O/10% D_2_O, 0.4 mM imidazole, 25 mM NaH_2_PO_4_/Na_2_HPO_4_, pH 7, and transferred to a 5 mm Bruker NMR tube.

### 2.4. Acquisition and Processing of ^1^H NMR Spectra

Acquisition of one-dimensional (1D) ^1^H NMR spectra of the retina metabolite samples was conducted using a Bruker 600 MHz (^1^H Larmor frequency) AVANCE III solution NMR spectrometer equipped with a 5 mm triple resonance (^1^H, ^13^C, ^15^N) liquid helium-cooled cryoprobe, Top-Spin software (Billerica, MA, USA, Bruker version 3.2) and an automatic sample loading system (SampleJet). The probe was held at a constant temperature of 300 K, and a gradient-based water suppression “zgesgp” pulse sequence [20,21,30,31,32] was employed to acquire the data, using a 12 ppm spectral window, 2048 scans, and 65 K data points. The dwell time between data point acquisition was set to 69 µs, resulting in a total FID acquisition time of 4.5 s. The recovery delay time (D1) between acquisitions was set to 2 s, yielding a total relaxation recovery delay of 6.5 s between scans. The Topspin (Billerica, MA, USA, Bruker version 3.2) software was used to reference chemical shifts to the most up-field 0.0 ppm DSS peak and to apply spectral phase correction, as previously reported [20,21,30,31,32,33]. Topspin was also used to execute baseline correction and to apply a 0.80 Hz line broadening EM (exponential multiplication) function, prior to Fourier transformation, and to yield preprocessed ‘1r’ NMR data files.

### 2.5. Chenomx Spectral Signal Annotation and Quantitation

Additional processing of the NMR spectra and metabolite identification (i.e., metabolite ID) and quantification were performed using the Chenomx^TM^ NMR Suite software (version 8.4; Chenomx Inc., Edmonton, AB, Canada). Using methods previously reported [33,34], the baseline of the imported ‘1r’ file was further adjusted by applying an automatic cubic spline function (Chenomx Spline) and manually adjusting and placing breakpoints using the Processor module of Chenomx. This resulted in flat and well-defined spectral baselines while minimizing the distortion of spectral signal intensities for metabolite quantification. Chemical shift referencing was achieved using DSS (as mentioned above), and the NMR signals of imidazole were used to correct for small discrepancies in chemical shift values arising from slight pH differences between samples.

The Profiler module of Chenomx was employed to annotate and quantify polar metabolites present in retina metabolite samples. Reference spectra from the Chenomx small molecule spectral database for 14.1 T magnetic field strength NMR spectrometers and the human metabolomics database (HMDB) [35,36] were used to fit ^1^H chemical shifts, ^1^H spectral splitting patterns, and signal intensities for each metabolite identified in the 1D ^1^H NMR spectra of the metabolite mixtures recovered from the FD mouse retinae. NMR signals were manually fit by visually matching the overall shape and intensity of the NMR resonances of standard compounds present in the Chenomx and HMDB databases, allowing for optimal matching of NMR chemical shifts, spectral pattern fit, and signal intensity [37]. Quantitation of identified metabolites was achieved using the intensity of the most upfield DSS (0.25 mM) signal as reference. A characteristic 1D ^1^H NMR spectrum collected on the retinae of a P60 FD mouse, with annotated metabolites labeled, is included as an illustration in the Supplementary Materials (Figure S1).

### 2.6. NMR and Statistical Analyses

Tables of identified and quantified metabolites were exported from Chenomx as µM concentrations. Normalization of the datasets by Logarithmic transformation (log_10_) and subsequent univariate and multivariate statistical analyses were carried out using MetaboAnalyst v 5.0. (Edmonton, AB, Canada) [38,39]. Retinae were evaluated and determined to be of comparable size and mass. Metabolite level differences between the CKO and controls were evaluated using Principal Component Analysis (PCA), hierarchical clustering analysis and heatmap visualization, *t*-tests, and a volcano plot analysis that accounts for both fold change and two sample *t*-test *p* values.

### 2.7. Extraction of Mouse Retinae for Dopamine Isolation

Mice were euthanized as previously described (Section 2.2), and the methods described in Pérez-Fernández et al. [40] were adapted for MS analysis of dopamine levels in Pax6 mouse retinae. Retina pairs from P30 mice were dissected in PBS, collected in a 1.5 mL microcentrifuge tube, flash frozen, and stored at −80 °C until further processed. Upon removal from storage at −80 °C, each sample was immediately homogenized with a micro-pestle while still frozen. Then, 40 µL of the extraction mixture (0.5 mM ascorbic acid and 1% formic acid) was added, and the sample homogenized further for 1 min and placed on ice. Following manual homogenization, all samples were sonicated for 5 min and then centrifuged at 14,000× *g* for 5 min. To prevent oxidation of dopamine, the resulting supernatant was subject to MS analysis within 5 h of the metabolite extraction procedure.

### 2.8. UHPLC/Q-TOF-MS Analysis for Relative Dopamine Quantitation

UHPLC/Q-TOF-MS (ultra-high performance liquid chromatography-quadrupole time-of-flight mass spectrometry) analysis of the FD retina samples was conducted at the Montana State University Mass Spectrometry Facility. An Agilent 1290 LC coupled to an Agilent 6538 Q-TOF was employed. UHPLC used an Agilent Eclipse Plus C18 chromatography column (1.8 µm particle size, 2.1 × 50 mm) at 50 °C with a flow rate of 0.6 mL/min and a sample injection volume of 5 μL. Water (0.1% formic acid) and acetonitrile (0.1% formic acid) represented the mobile phase solvents A and B, respectively. Initially, mobile phase conditions of 99% A and 1% B were held constant for 2 min, followed by a linear gradient from 1% to 98% B over 6 min, a hold of 1.25 min at 99% B, then a return to the initial condition for a 2.75 min re-equilibration, resulting in a total MS run time of 12 min. The LC eluent was ionized using electrospray ionization in positive mode, and the Agilent 6538 Q-TOF was operated at 2 Hz over a mass range of 50–1000 *m*/*z*. A dopamine standard (Sigma-Aldrich CAS-No: 62-31-7) was used to verify the *m*/*z* value and retention time. Relative quantitation was achieved using the Agilent MassHunter Quantitative Analysis for Q-TOF (v B.07.00/build 7.0.457.0) software, and values were reported as extracted ion chromatogram (EIC) area. Retinae from P30 CKO mice were evaluated based on their concentration of dopamine, relative to age matched litter controls with two separate MS experiments. Due to batch effect variability that can be introduced based on separate MS experiments, the data were not combined and is labeled as Run 1 and Run 2. The difference in dopamine levels in the CKOs compared to controls was assessed with a two-tailed *t*-test for equal variance with a *p*-value threshold <0.05.

### 2.9. Retinal Histology for Flat Mount Preparations

P7 mice were euthanized by isoflurane exposure and rapid decapitation. P30 and P90 mice were euthanized as previously described in Section 2.2. The temporal surface of the eye was marked with green dye (Ketchum Manufacturing) prior to enucleation. The tissue was fixed in 4% paraformaldehyde (PFA) at room temperature (RT) for 1 h. The retinae were excised, and the temporal region was marked by a small 15-degree cut. The tissue was permeabilized in 1× PBS containing 0.5% Triton X-100 for 30 min at RT with gentle shaking. Non-specific binding was blocked using 1× PBS containing 0.5% Triton X-100 and 5% bovine serum albumin (BSA) at RT for 3 h with gentle shaking. All retinae were incubated for 2 nights at 4 °C with a primary antibody against tyrosine hydroxylase (TH) (rabbit anti-Tyrosine Hydroxylase, Millipore, Catalog No. AB152) or against RNA-binding protein with multiple-splicing (RBPMS) (guinea pig anti-RBPMS, PhosphoSolutions, RRID_AB_249226) diluted in 1% BSA, 0.1% Triton X-100 in 1X PBS, followed by overnight incubation at 4 °C with a secondary antibody (Donkey anti-rabbit 488, RRID_AB_2535792, or Donkey anti-guinea pig 647, RRID_AB_2735091; Thermo Fisher Scientific, Waltham, MA, USA). Four identical radial cuts flattened the tissue into four lobes, leaving the central region intact. The tissue was mounted with the ganglion cell layer (GCL) oriented facing up on microscope slides using ProLong^TM^ Gold Antifade Mountant (Invitrogen, Cat# P36930, Waltham, MA, USA).

Retinal flat mounts were imaged using a Leica DMi8 THUNDER imaging system using the navigator spiral scan function with a 20× objective under brightfield illumination with false color. Confocal images of flat-mount retinae were obtained using Lecia TSC SP8 with a 20× oil immersion objective with a resolution of 1024 × 1024. A single image 1mm from the peripheral edge of the tissue in each retinal quadrant was obtained.

Retinal images were processed using ImageJ plugin: Fiji [41]. The total TH+ cells in each retina were counted manually. All images were randomized, ensuring that the experimenter was blind to the genotype. The data were reported as the total number of TH+ amacrine cells per retina. For pairwise comparisons, unpaired two-tailed *t*-tests were used with a *p* < 0.05 indicating significance. All data were expressed as mean ± SEM. Graphs were made using GraphPad Prism (version 9). Column data are plotted as scatter dot-bar plots to show variability, and n is the sample size for each dataset.

## 3. Results

### 3.1. Retina Metabolomic Differences Emerge by 2 Months of Age

A total of 14 retina pairs were collected from P7 mice (n = 7 CKO, n = 7 controls), and 28 metabolites were identified and quantified using NMR (Table S1). Similarly, 17 retina pairs were collected from P60 mice (n = 9 CKO, n = 8 controls). The same 28 metabolites identified in the P7 dataset were measured in the P60 dataset (Table S2 and Figure S1). These two time points were selected because CKO and control mice are born with equal numbers of RGCs, and then RGCs in the CKO die progressively postnatally, becoming significantly reduced compared to controls by P30 [10,27].

**Figure 1:**
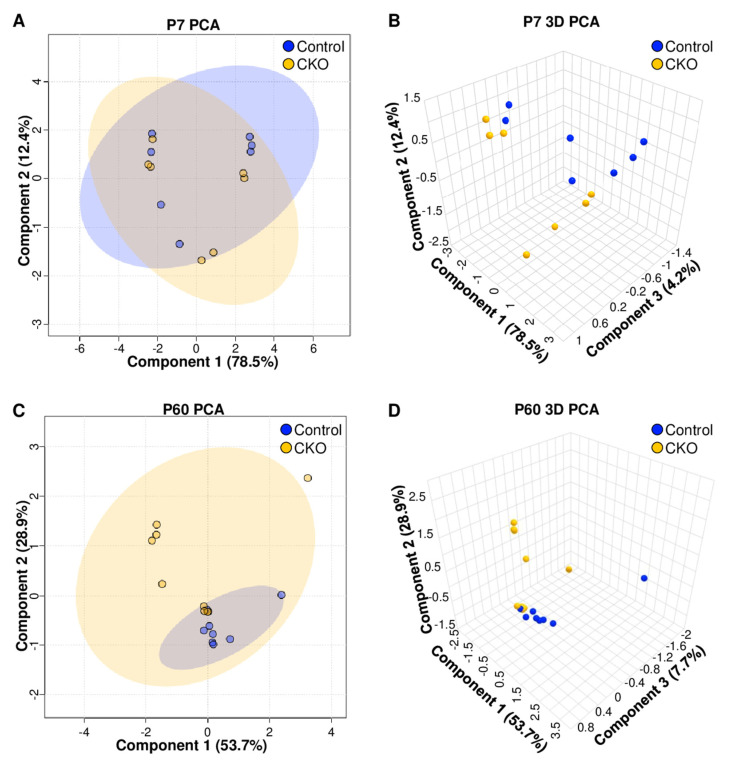
Multivariate principal component analyses (PCA) of the metabolic profiles of P7 and P60 retinae indicate that the CKO group (gold) separated from the control group (blue) by P60. Panels (**A**,**B**) present the 2D- and 3D-PCA scores plots of retinae at P7, while panels (**C**,**D**) present the 2D- and 3D-PCA scores plots for retinae collected at P60. (**A**) 2D PCA scores plots of P7 retinae show a complete overlap of samples for both groups, indicating any differences in metabolomic profiles were not sufficiently distinct to separate the CKO from controls. (**B**) With the addition of a third component, metabolite patterns of the CKO retinae appeared to slightly cluster separately from those of control samples in the 3D PCA scores plot of the P7 retinae; however, principal component 3 only accounted for 4.2% of the variance, indicating a marginal group separation. (**C**) 2D PCA scores plots of the metabolic profiles of P60 retinae revealed that the CKO samples were more variable than controls, but clustered separately from controls when examining the resulting 3D PCA scores plot (**D**). Each dot represents each sample generated by collecting the two retinae of each mouse.

Differences in metabolic profiles between CKO and control retinae from P7 and P60 retinae were first assessed using an unsupervised multivariate PCA analysis (Figure 1). Two-dimensional (2D) PCA scores plot shown in Figure 1A for P7 retinae revealed intermingled clustering of all datapoints, with no clear separation of the CKO and control groups. Three-dimensional (3D) PCA scores plot analysis of the metabolite profiles of the P7 mouse samples revealed a marginal though noticeable separation between the P7 CKO and control mouse groups (Figure 1B), indicating a potential trend emerging in metabolite differences that makes it possible to separate the two mouse groups based on distinct retina metabolite patterns.

PCA analysis of the P60 retina samples (Figure 1C,D) revealed a clear difference in metabolite patterns between the CKO and control groups, as assessed by a tight clustering of the control samples and reduced overlap between CKO and control groups in 2D PCA scores plots (Figure 1C), with group separation being most pronounced along principal component 2 (PC2). When including a third principal component, which led to 90% of the variance being accounted for by PC1–PC3, the resulting 3D PCA scores plot (Figure 1D) demonstrated a distinct clustering of the CKO group separate from the control group. This PCA analysis thus indicated that by P60, retina metabolome differences were sufficiently distinct to clearly separate CKO retina samples from control samples.

**Figure 2:**
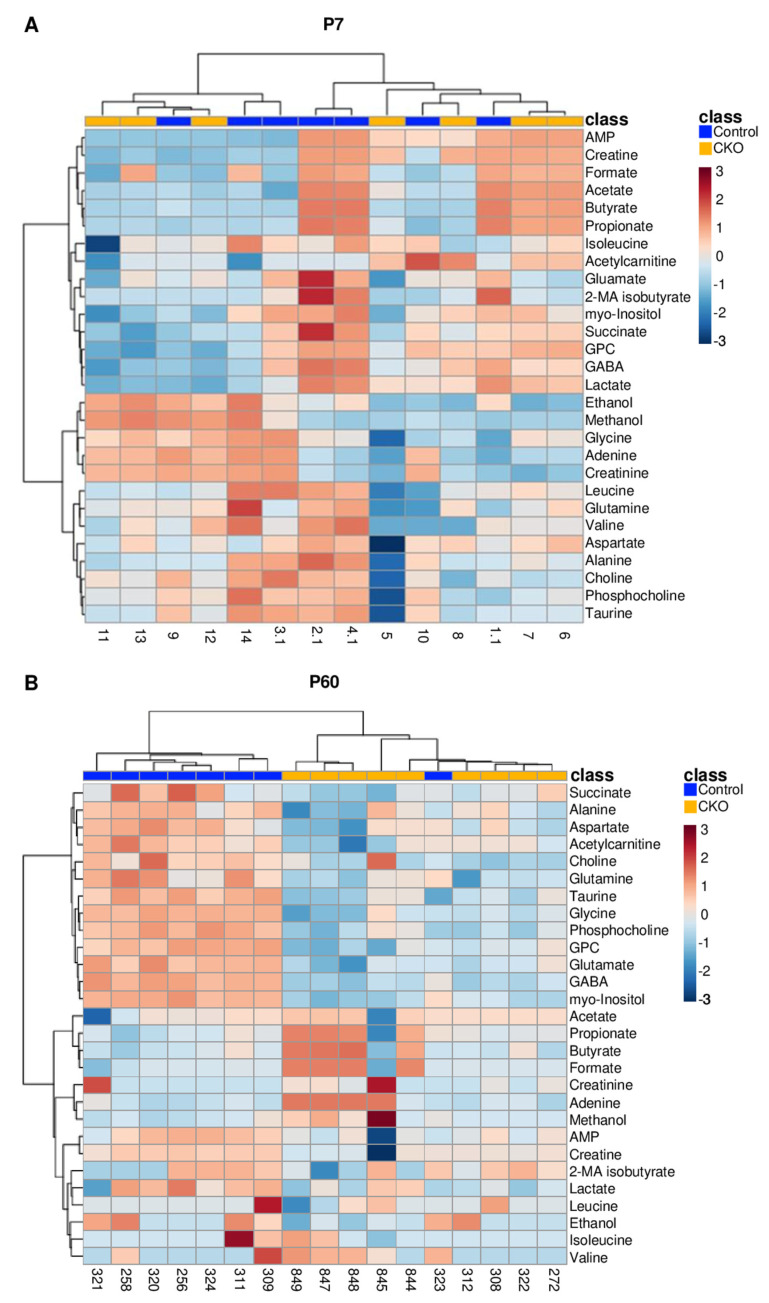
Hierarchical clustering analysis (HCA) and heatmap visualizations of (**A**) P7 and (**B**) P60 CKO (gold) and control (blue) retina samples. The red-blue color spectrum represents the scaled abundance of each metabolite, with dark red and blue indicating high and low abundance, respectively. No distinct clustering based on metabolite levels and genotype were observed until P60. Abbreviations: AMP, adenosine monophosphate; 2-MA isobutyrate, 2-methylamino isobutyrate; GPC, glycerophosphocholine; GABA, gamma-aminobutyric acid.

Differences in the metabolite profiles of CKO and control mouse retinae were further assessed through a hierarchical cluster analysis (HCA) and visualized using heatmaps. By P60, samples from the CKO group clustered more closely together (based on their characteristic metabolite level patterns) compared to the control group, while this distinction was not discernable at P7 (Figure 2). These results are consistent with observations resulting from the PCA analyses of the P7 and P60 mouse groups. The modest differences in metabolite levels measured in CKO and control retina samples at P7 were not sufficiently significant to separate the two mouse groups by genotype at that time point (Figure 2A).

At P60, the CKO samples separated from the controls, except for sample 323 whose metabolite profile was closer to that of the CKOs. We did not have a good rationale to remove sample 323, and inclusion of the datapoint did not change the significance of the results, so it was kept in the dataset. At P60, the heatmap indicates that many metabolites were lower in concentration in the CKO retina samples relative to control samples, as shown by the blue and red color blocks associated with each cluster. Metabolites with greatest level differences in the CKO mice compared to controls included succinate, alanine, aspartate, acetylcarnitine, choline, glutamine, taurine, glycine, phosphocholine, glycerophosphocholine (GPC), glutamate, gamma-aminobutyric acid (GABA), and myo-inositol, all with lower concentrations in the CKO group, relative to the control group.

**Figure 3:**
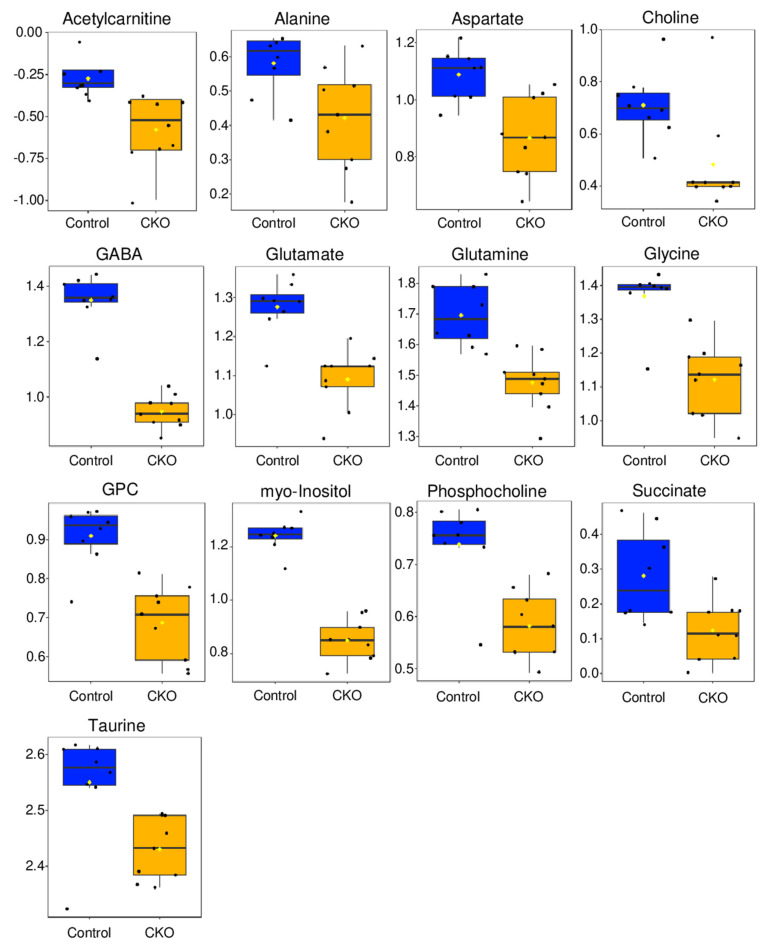
The two-sample *t*-test of the P60 retinae metabolomic profiles identified 13 metabolites that significantly discriminated the CKOs from controls. The *t*-test parameters assumed equal variance and employed an FDR corrected *p* value threshold < 0.05. The *y*-axis is relative normalized concentration; boxes represent the 1st and 3rd quartile, the black line is the median, yellow dot is the mean, and each black dot represents a single sample.

Conducting an unpaired, two-tailed *t*-test revealed no significant differences between the metabolites measured for the CKO and control retina samples at P7, but a *t*-test of the metabolite concentrations of the P60 samples identified 13 metabolites with levels that were significantly (FDR *p* < 0.05) altered between the CKO and control groups, all being lower in the CKO group (Figure 3). The 13 metabolites with significantly altered levels included acetylcarnitine, alanine, aspartate, choline, GABA, glutamate, glutamine, glycine, glycerophosphocholine (GPC), myo-inositol, phosphocholine, succinate, and taurine (Figure 3).

A volcano plot analysis of differential metabolite levels was performed for the P60 retina samples (Figure S2, Table S3). This analysis was used to examine which metabolites were most altered in concentration between the CKO and control groups based on fold change (FC) concentration differences and excluded metabolites that did not reach FDR-corrected *p* < 0.05 through *t*-test evaluation (Figure S2, Table S3). This analysis revealed that the levels of myo-inositol and GABA were 2.5 times lower in the CKO retinae compared to controls. The lower levels of glycine, glycerophosphocholine, glutamate, glutamine, aspartate, acetylcarnitine, and choline ranged between 1.7 and 1.5 folds for the CKO retinae compared to controls, while all other metabolites identified in the two-sample *t*-test were reduced by a FC ≤ 1.5.

Overall, these analyses indicated no statistically significant differences in metabolite levels at P7 but identified significant metabolite level changes for the CKO retinae compared to control samples at P60, with myo-inositol and GABA being the lowest in concentration in the CKO group compared to the control group. Metabolites that most discriminated between the P60 CKO and control groups belonged to diverse classes including amino acids, phospholipid metabolism constituents, inhibitory and excitatory neurotransmitters, and metabolites involved in retina energy (ATP) metabolism.

### 3.2. Alterations in Dopaminergic Amacrine Cell Population in CKO Retinae

Dopaminergic amacrine (DA) cells are modulatory retinal interneurons involved in key visual processes such as light adaptation and color vision [13,14]. In the mouse retina, DA cells are strictly positioned within the inner nuclear layer (INL) and extend processes to other retinal layers; dopamine receptors are distributed throughout the retina supporting a modulatory role for DA cells in the retina [42]. Dopamine transmission via DA cells utilizes volume transmission, allowing for the pan-retinal modulatory affect.

**Figure 4:**
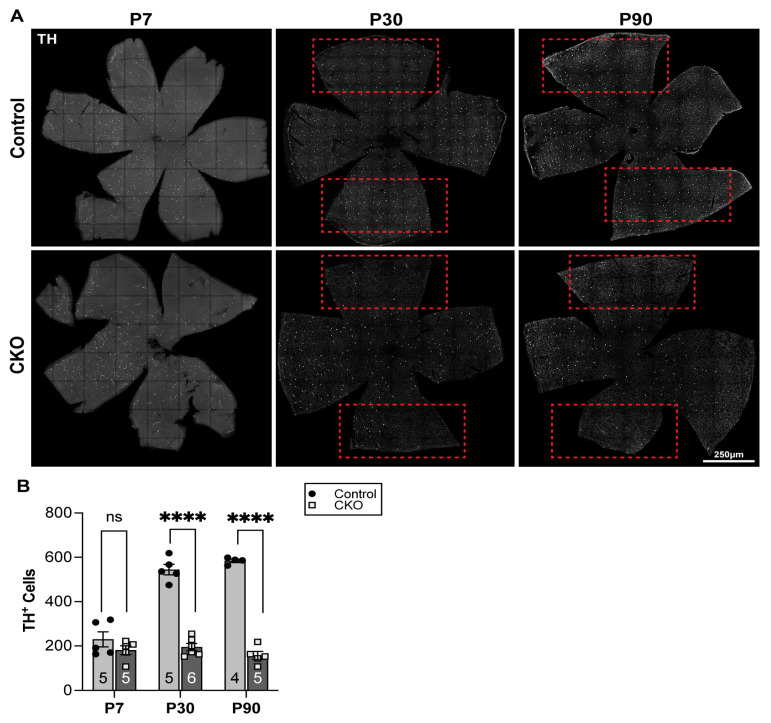
TH+ amacrine cells failed to increase in number as CKO mice mature. (**A**) Representative retina flat mount stained with anti-TH at three different ages (columns) demonstrating increases in cell number in control (top panels) but not in CKO (bottom panels) mice. Red boxes indicate regions with the most prominent reduction in the TH cells in the CKO and corresponding regions in the control retinae. Scale bar, 250 µm. (**B**) Quantification of TH+ amacrine cells from control mice compared to CKO mice. TH+ cells were counted in the entire retina. ns = 0.2545, **** *p* < 0.0001, unpaired *t*-test. Data are shown as average ± SEM, and each data point represents an individual retina. The sample size for individual groups is represented by n within each bar.

Interestingly, FD mouse models have reduced numbers of tyrosine hydroxylase (TH)-positive neurons in the nodose-petrosal ganglia [43] and in the dorsal root ganglia [44], further suggesting that these neurons may be reduced in the FD retinae and that their deficit may contribute to the progressive visual decline of FD patients. Based on these data, including the retinal degeneration characterized in FD patients and finding reduced levels of GABA at P60 (Figure 3 and Figure S2, Table S3), which was primarily expressed in a subset of amacrine cells, we quantified the number of the DA cells. CKO and control retinae at P7, P30, and P90, were immunolabeled with an antibody to tyrosine hydroxylase (TH), as this is the rate-limiting step in dopamine synthesis [45]. Differences in the number of TH-positive cells in CKO retinae compared to littermate controls were observed at the later time points examined (Figure 4). At P7, before mice had opened their eyes and all amacrine cells had been generated, there was no difference in the number of TH-positive cells between the CKO and control (Figure 4A,B). However, by P30, when substantial RGC death had occurred (see Supplementary Figure S3 and Ueki et al., 2018 [10]), significantly fewer TH-positive amacrine cells were present in the CKO (Figure 4A,B; **** *p* < 0.0001). This trend continued until P90, with a similar number of TH-positive cells in the CKO being significantly reduced in the CKO compared to the control (Figure 4A,B; **** *p* < 0.0001). Interestingly, the number of TH-positive cells in the CKO retinae remained consistent across time points, suggesting that their precursors fail to differentiate, rather than die.

### 3.3. Reduced Dopamine Levels at P30 in CKO Retina

**Figure 5:**
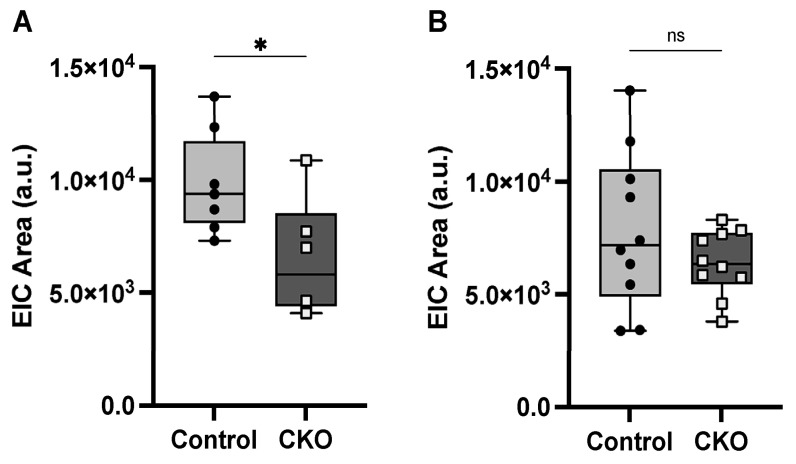
Retina dopamine levels were reduced ~25–35% in Elp1 CKOs compared to controls, as measured by LC-MS. Run 1 (**A**) and Run 2 (**B**) indicate the results from the two separate MS experiments conducted and depict the relative quantitation of dopamine levels as determined from measurement of the area under extracted iron chromatogram (EIC) LC-MS signals. Each data point represents both retinas of an individual mouse. * *p* = 0.023.

Dopaminergic amacrine (DA) cells are the primary source of dopamine in the retina [38]; thus, relative dopamine levels in CKO and control retinae were measured using UHPLC/Q-TOF-MS to determine whether the significant reduction in DA cells was associated with lower dopamine levels. Two MS experiments (Run 1 and Run 2) were conducted as described in Section 2.8, and data were analyzed separately due to batch variability. Dopamine levels at P30 in Run 1 were significantly (*p* value < 0.05) reduced by ~35% in CKO retinae compared to controls (*p* = 0.023) (Figure 5). Though Run 2 did not reach significance (*p* < 0.05), a ~25% reduction in dopamine levels was still observed in CKO retinae, relative to the amount measured in the control retinae (Figure 5). Overall, while there was variability in the dopamine levels between the CKO and control retinae, we can confidently report a ~25–35% reduction of total dopamine levels in the CKO mouse retinae compared to control samples at P30. These findings are supported by the consistent reduction in the number of DA cells in the CKO mice as they matured.

## 4. Discussion

Here, we report a metabolomics analysis of the retina in a *Pax6-cre*; *Elp1^LoxP/LoxP^* CKO mouse model for FD. This study was conducted to better understand how metabolism may contribute to the optic neuropathy observed in FD patients. In addition, we investigated the dopaminergic amacrine cell population via immunohistochemistry and targeted mass spectrometry (MS) in the FD mouse retina. Metabolic alterations were observed to occur over time and in tandem RGC degeneration, which was marked by significant changes in taurine levels, Cahill and mini-Krebs cycle intermediates, phospholipid metabolism, and metabolites associated with maintaining proper mitochondrial function. The data demonstrated that *Elp1* CKO mouse retinae had a 25–35% reduction in dopamine levels at P30, which was accompanied by an overall reduction in TH+ cell numbers compared to controls beginning at P30.

FD is a highly debilitating neurodevelopmental and neurodegenerative disease, with a ubiquitous clinical phenotype of progressive visual decline that begins by 20 years of age [9]. Previous studies that have included patient optical coherence tomography (OCT) imaging and retina pathology from FD patients and/or mouse models have reported evidence of mitochondrial dysfunction, reduced ATP production, neuronal stress, and secondary cell types affected by loss of Elp1 [9,10,27]. The role of mitochondrial dysfunction and reduced energy production in the CKO retinae have necessitated a deeper understanding of how metabolic impairments affect the function of the retina in FD. The data reported here on retina metabolic changes in FD support our recently published work demonstrating significant alterations in the polar metabolite profiles of FD patients’ serum and fecal samples [20,21]. Collectively, our data highlight the interconnection between the FD disease state and central metabolism and identify the metabolic processes that may promote and/or are associated with neurodegeneration in FD.

### 4.1. ^1^H NMR Data Indicate That Metabolic Disturbances Correlate Temporally with Elp1-Induced RGC Degeneration

At postnatal day 7 (P7), the metabolome of retinae from FD mice could not be separated from the control mice as differences in polar metabolite patterns were small and not statistically significant (Figure 1A,B and Figure 2A). Metabolite pattern trends appeared to be emerging based on slight group differentiation observed in the P7 3D PCA plot (Figure 1B), though when evaluated by *t*-test, no metabolites were significantly different based on an FDR corrected *p* value < 0.05. Conversely, by P60, the metabolome of CKO retinae diverged from that of controls, enabling the separate clustering of the CKO group from the control group in 2D and 3D PCA scores plots (Figure 1C,D). Additional analysis using a two-sample *t*-test identified 13 metabolites that had significantly different levels (FDR *p* ≤ 0.05) in the CKO retinae compared to controls. These data suggest that the onset of RGC decline is associated with metabolic alterations. Metabolites that statistically discriminated between the retina metabolome of CKO mice and that of control mice at P60 (Figure 3) were further evaluated based on their associated metabolic pathways and relevant cellular functions specific to the retina.

The retina is the most energy-demanding neuronal tissue [22,25] and thus requires efficient metabolic pathways to meet its energetic (ATP) needs. Consequently, retinal cells are highly sensitive to disturbances in energy supplies and oxidative stress [26,46]. RGCs, which extend long axons to connect with their CNS targets, rely critically on proper mitochondrial function for survival, and disruption in oxidative mitochondrial metabolism within RGCs has been linked to other optic neuropathies in addition to FD, including glaucoma, Leber’s hereditary optic neuropathy (LHON), and dominant optic atrophy (DOA) [23,24,25]. In this study, taurine, and other metabolites involved in healthy retina mitochondrial function and energy production, were identified as being key markers of CKO retina pathology when compared to control retinae at P60.

### 4.2. Taurine Is Fundamental for Mitochondrial Electron Transport Chain (ETC) Complex 1 Function and RGC Health

In our previous metabolomics analysis of FD patients, taurine levels were systemically low in the sera of FD patients and high in their stool samples [20,21]. Herein, taurine levels were also significantly decreased in the FD mouse retinae at P60 (Figure 3). Compared to other tissues, the retina contains high levels of taurine, and deficits in taurine cause loss of functional photoreceptors, RGC degeneration, and optic neuropathy [47,48,49,50,51]. The mechanism by which reduction of taurine level induces retinal damage is thought to occur via impairment of mitochondrial function, including reduced complex 1 electron transport chain (ETC) activity and oxygen consumption (reduced respiration), and increased reactive oxygen species (ROS) production [52,53]. Analysis of an FD patient muscle biopsy revealed reduced ETC complex 1 function in FD (F. Lefcort, Personal Communication), a deficit which has been similarly observed in Pax6 mouse retinae at 2.5 months of age [10]. Mutations in genes that impair complex 1 function cause LHON and DOA [23,24]. As such, our observation of reduced levels of taurine in CKO retinae presents an interesting overlap between taurine-deficient phenotypes and a reduction in Elp1, as both cellular states result in mitochondrial impairment through a similar mechanism of mediating wobble uridine translation and ultimately reducing ETC protein translation efficiency [54,55]. Elp1, as the key scaffolding subunit of the Elongator complex, modifies the wobble uridine of tRNA for the proper translation of codons for glutamate, glutamine, and lysine [54]. Meanwhile, taurine conjugation also enables the smooth translation of codons for mitochondrial leucine and lysine via tRNA wobble uridine modification in the mitochondria [52,55].

Reduction of both Elp1 and taurine in the CKO retina could thus act in concert, exacerbating deficiencies in wobble uridine modification and translation. Interestingly, the wobble uridine modification facilitated by taurine conjugation is necessary for the synthesis of mitochondrial proteins cytochrome b, ND5, and ND6 (NADH-ubiquinone oxidoreductase chain 5 and 6) [52,55], the latter two being the same genes that are mutated in LHON and DOA [23,24]. Moreover, all are responsible for maintaining proper complex 1 function, minimizing ROS production and cellular stress, and preventing the breakdown of mitochondrial structure and function. Improving mitochondrial function in dorsal root ganglia (DRG) neurons improved neuronal survival in FD mice [56], indicating that improving mitochondrial health could be a promising target to prevent RGC loss [10].

Taurine has a direct role in protecting mitochondrial function, and taurine dietary supplementation studies have shown that taurine can mitigate metabolic impairments, mitochondrial dysfunction [53], and retinal degeneration [49]. Taurine is thus a promising metabolite for future studies on its involvement in FD retinal pathology and may be a good candidate metabolite for therapeutic interventions focused on mitigating the progressive optic atrophy observed in FD patients, as well as that of LHON and DOA.

### 4.3. Alterations in the Levels of Metabolites Involved in the Cahill (Alanine-Glucose) and Mini-Krebs Cycles Point to Energetic (ATP) Deficits in CKO Retinae

Retinal tissue, like the brain, derives its metabolic energy predominantly by glycolysis [22,25]. Though glucose is the preferred substrate of the retina for energy production, like the brain, there is evidence that other substrates such as lactate or certain amino acids can be an energy source.

**Figure 6:**
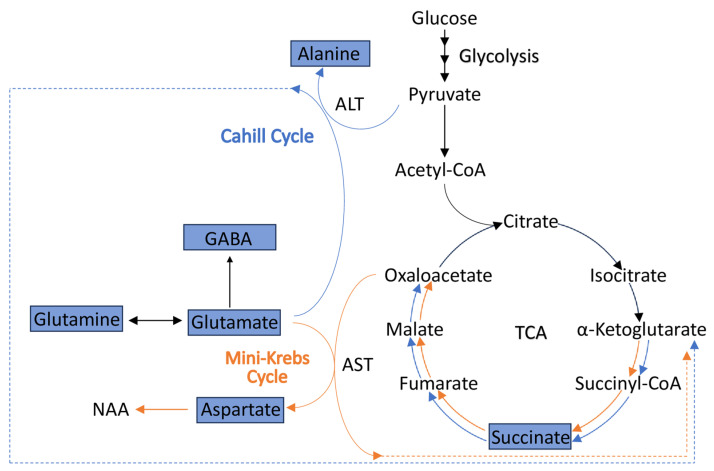
An illustration of how the Cahill and mini-Krebs cycle both contribute to rapid energy production in the retina. The Cahill and mini-Krebs cycles are shown in blue and orange, respectively. Key steps in each cycle are depicted, with ALT initiating the Cahill cycle by converting pyruvate and glutamate to alanine and α-ketoglutarate, respectively, which can then enter the TCA cycle. In a similar manner, glutamate and oxaloacetate can be converted to aspartate and α-ketoglutarate, via AST function, thus entering the TCA cycle as well. Both pathways provide means by which ATP, NADH, GTP, and FADH_2_ can be generated at an efficient rate in the absence of glycolysis coupled to aerobic catabolism via the TCA cycle in the retina [22]. The characteristic signature metabolites that indicate Cahill and mini-Krebs cycle activity include alanine (Cahill) and NAA (mini-Krebs cycle) [22]. Blue boxes indicate metabolites with significantly lower concentrations measured in the Pax6 CKO retina compared to controls. Abbreviations: ALT, alanine transaminase; AST aspartate aminotransferase; NAA, n-acetyl aspartate.

Metabolic pathways of the brain are characterized by the reliance on glucose for glycolysis, as well as TCA cycle activity that is fueled by ketone catabolism and the malate-aspartate shuttle that supplies substrates for replenishing the TCA cycle intermediates [57]. Recent investigations into the energetic needs of the retina have identified an uncoupling of glycolysis and the TCA cycle through the “mini-Krebs” (6-step TCA) cycle and the alanine-glucose Cahill cycle (Figure 6), which appears to take place in photoreceptors and Müller glia [22], utilizing similar metabolic pathways as those described for the brain [57].

In the Cahill cycle, pyruvate produced from glycolysis is transaminated to alanine, thus converting glutamate to α-ketoglutarate, which can enter the TCA cycle [22]. In the mini-Krebs cycle, metabolites enter the TCA cycle via the conversion of glutamate and oxaloacetate to α-ketoglutarate and aspartate, respectively, a reaction mediated by aspartate amino transferase. Glutamate is a key metabolite for both pathways. In Müller glia, and presumably other retinal cell types, glutamate can be derived from glutamine or absorbed from the extracellular space [22]. An additional metabolic fate of glutamine, beyond its role in energy metabolism, is its conversion to GABA (gamma amino-butyric acid), the most abundant inhibitory neurotransmitter, which is used by DA cells [15].

The reduced levels of alanine, glutamine, glutamate, and aspartate found in P60 CKO Pax6 retinae suggest a potential deficit or depletion of these key energy-producing substrates. GABA levels were also reduced in the P60 CKO retinae, suggesting the lower levels of glutamate are not a result of GABA production. A third pathway that uncouples glycolysis and the TCA cycle, similarly to the Cahill and mini-Krebs cycle, is the Cori (lactate-glucose) cycle, which reduces pyruvate to lactate to maintain anaerobic glycolysis by regenerating NAD+, though this pathway is not as efficient in the retina at maintaining ATP production as the Cahill and mini-Krebs cycle [22]. Lactate levels were measured in the CKO and control retinae, though no significant differences in lactate levels were observed, suggesting that the Cori cycle may not be substantially upregulated. The fact that succinate levels were not found to be elevated in CKO Pax 6 retinae suggests that the TCA cycle is indeed active and could be fueled through the shuttling of intermediates through the mini-Krebs cycle (Figure 6).

The function of the Cahill and mini-Krebs cycles have not been explored in RGCs, but instead have been characterized in photoreceptors and Müller glia, suggesting the observed metabolic changes may be attributed to dysregulated metabolic function in cell types other than RGCs, even if other retinal cell types are not dying. Retinal ganglion cells have been the only cell type reported to die in the Pax6 FD mouse model, though other cell types seem to have impaired functions [10]. We now show that an additional neuronal subtype is affected by the loss of Elp1. Future studies are needed to determine if the cells fail to proliferate or differentiate to generate the full complement of TH+ cells. Photoreceptors have died in other FD models, although at a much later age than the P60 timepoint used in this study with Pax6 FD mice [9], and there is no evidence that photoreceptors are reduced in the Pax6 FD mouse model [10]. These results indicate that the metabolic changes identified in this study are either indicative of alterations specific to RGCs that should be further explored, or these data present evidence of alterations associated with cell types other than RGCs that may promote RGC degeneration indirectly through the dysregulation of cell function.

### 4.4. Reduced Levels of Membrane Phospholipid-Associated Metabolites Myo-Inositol and Choline Are Associated with Retinopathy

Both myo-inositol and phosphocholine were significantly reduced in the Elp1 CKO retina (Figure 2 and Figure 3). Myo-inositol is the most abundant of nine isoforms of inositol and is comprised of a six-carbon ring structure, each with an attached hydroxyl group [58]. Myo-inositol is commonly found in tissues with high glucose utilization [58], including the retina, and is a marker for glial cell proliferation [59].

Myo-inositol is present as a result of dietary absorption and endogenous synthesis. Glucose-6-phosphate is used to synthesize myo-inositol phosphate, which is then converted to myo-inositol through L-myo-inositol-1-phosphate synthase and myo-inositol-1-phsophate catalysis. This synthesis occurs in the liver, kidneys, and brain [58], but myo-inositol concentrations are primarily maintained in neural tissue by active cellular uptake that can be inhibited by elevated blood glucose levels. Reduced levels of cellular myo-inositol result in abnormal phospholipid metabolism of phosphoinositides [60], which make up a substantial component of retinal cell membranes [61]. Additionally, reduced levels of myo-inositol can impact cholinergic transmission by improperly mediating the assembly and disassembly of microtubules that are involved in neurotransmitter release [60]. The cholinergic nervous system has been greatly impacted in FD and FD mouse models [62,63], suggesting a mechanism by which reduced levels of myo-inositol could be exacerbating dysfunction of the cholinergic system in FD.

Within the retina, the distribution of phosphatidylinositol is ubiquitous in retinal membranes of cell types other than rod outer segments and the retinal pigment epithelium [61]. Phosphoinositides are important for numerous cellular functions, including development, proliferation, differentiation, membrane excitability, exocytosis, phagocytosis, cell motility, and the detection of extracellular signals, as the enzymes mediating these functions contain highly specific phosphoinositide-binding domains. Thus, the pathways regulated by phosphoinositides are essential for the function and health of the retina and for mediating cellular responses to a disease state [61]. Given the need for downstream products of myo-inositol metabolism for numerous enzyme functions, restoring myo-inositol levels in FD retinae may be beneficial to ensure proper protein synthesis, DNA repair, and survival signaling through PTEN activity and other phosphoinositide-dependent enzymes [64]. This observation is particularly noteworthy given that expression of genes involved in DNA repair pathways is reduced in Elp1 CKO neurons in an FD mouse model [54].

Similarly to myo-inositol, choline is a precursor for another crucial phospholipid, phosphatidylcholine, and was reduced in Pax6 CKO retinae at P30 (Figure 3). In neurons, choline facilitates cellular homeostasis via two primary metabolic fates: phosphatidylcholine synthesis, and acetylcholine production. Phosphatidylcholine functions both as a primary cellular membrane constituent and as a storage molecule to maintain localized neural pools of choline for acetylcholine synthesis [65,66]. Both acetylcholine and an intermediate of phosphatidylcholine synthesis (citicoline) are crucial for the survival and viability of RGC cells [67], the cell types that die in FD [7,9,10].

**Figure 7:**
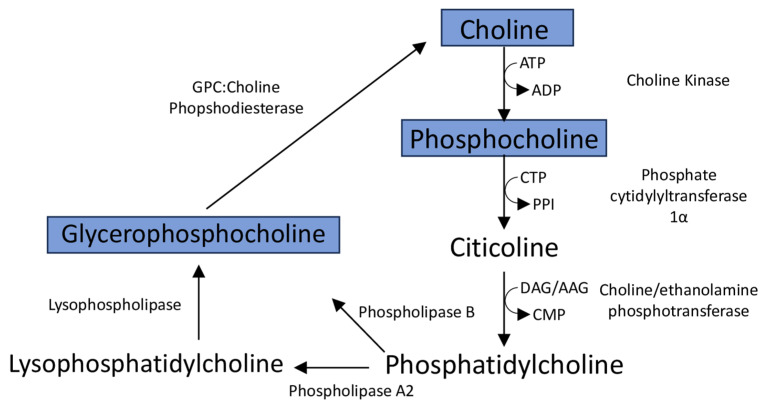
Phosphatidylcholine production and catabolism. Blue boxes indicate metabolites with significantly lower levels measured in the Pax6 CKO retina compared to controls. Abbreviations: ATP, adenosine triphosphate; ADP, adenosine diphosphate; CTP, cytidine triphosphate; PPI, inorganic pyrophosphate; DAG, diacylglycerol; AAG, alkyl-acyl-glycerol; CMP, Cytidine monophosphate; GPC, glycerophosphocholine.

Lower levels of choline, phosphocholine, and glycerophosphocholine were measured in the CKO retinae compared to controls. Choline and phosphocholine were the substrate and product, respectively, of the first reaction of the four-step Kennedy pathway of phosphatidylcholine synthesis (Figure 7). Glycerophosphocholine is a catabolic product of phosphatidylcholine and an intermediate in the de novo synthesis of choline. High cell membrane synthesis and an active Kennedy pathway are characterized by low intracellular choline and high phosphocholine levels [22,31].

Citicoline has been shown to promote acetylcholine and myelin production, restore mitochondrial function, to have anti-apoptotic effects, stimulate antioxidant activity, prevent neuronal cell death, and to counteract retinal nerve fiber layer (RNFL) thinning [67,68]. FD patients and mouse models have exhibited reduced myelination of axons, thinning of the retinal nerve fiber layer (RNFL), enhanced RGC death, and have deficits in mitochondrial integrity and function [2,6,7,8,10]. Though additional studies are needed to assess the function of the Kennedy pathway in Elp1 CKO retinae, it may be useful to evaluate levels of citicoline directly, as this metabolite represents a promising neuroprotectant to prevent or reduce RGC death and to limit visual decline in FD patients. In addition, citicoline has been shown to increase retinal dopamine concentrations [67], which were reduced in the CKO retinae as observed in our mass spectrometry (MS) analyses.

### 4.5. Alterations in Dopaminergic Cell Populations

This study provides the first evidence that a specific subtype of retinal neurons other than RGCs may also be affected by the loss of *Elp1*. Furthermore, these data might help explain the decline in visual acuity and color-vision deficits in FD patients [7,68]. Interestingly, it does not appear that the DA cells are dying because the number of TH-positive cells in the CKO does not decline, but rather, they do not increase in number over time as they do in the control. Since the progressive maturation of DA cells occurs over the first month postnatally, our finding suggests a failure to fully mature and differentiate into TH+ neurons in the absence of Elp1. These data are consistent with the targeted MS analysis that found a 25–35% reduction in dopamine levels at P30. All together, these data demonstrate that the DA cell population is reduced in the CKO retinae, which is also reflected in the reduced levels of available dopamine.

Amacrine cells are the most diverse cell type in the retina, and despite their differences, the majority of amacrine cells function as inhibitory interneurons, which can be divided into two groups based on whether the cells use GABA or glycine as their primary neurotransmitter. GABAergic cells and glycinergic cells can also express additional neurotransmitters [69,70,71], and DA cells are also GABAergic. Interestingly, the DA cells are one of the sparsest retinal neurons, comprising only 0.0001% of all retinal neurons.

Taken together, these observations indicate a potential problem with differentiation in a small subtype of neurons in the absence of Elp1. Additionally, lower amacrine cell numbers could lead to altered receptive properties of RGCs, which may result in additional visual processing dysfunction as seen in FD patients. Our initial study of retinal amacrine cells has yielded exciting results that warrant further investigation to better understand the impact of DA cell deficits in the FD retina, confirm potential defects in neuronal differentiation in the absence of Elp1, and elucidate yet another important biological function of Elp1 and the Elongator complex.

## 5. Conclusions

In conclusion, our NMR-based metabolomics analysis of *Pax6-Cre*; *Elp1^LoxP/LoxP^* CKO retinae demonstrated significant changes in polar metabolites associated with mitochondrial function, energy production, and phospholipid metabolism. Accompanying these data are the targeted dopamine MS analyses and histological examinations of a subpopulation of retinal amacrine cells, which showed significant reduction of both cellular dopamine levels and DA cells in CKO retinae compared to control retinae. These data have provided the first untargeted metabolic investigation of *Elp1* CKO retina, and demonstrate, for the first time, that a retinal cell population other than RGCs was directly impacted by the loss of *Elp1* in this FD mouse model. Of particular interest for future study are the metabolites taurine, myo-inositol, and small molecule constituents of the Kennedy pathway, including citicoline, as these metabolites show great promise as efficacious retinal neurotherapeutics which could be delivered in a non-invasive way. In addition, it would be worthwhile to further investigate why the levels of dopamine and numbers of DA cells are reduced in *Elp1* CKO FD retinae, as a clear mechanistic understanding of these phenomena could help better define the color vision decline observed in FD patients. Ultimately, results presented in this study have provided new knowledge on how metabolic deficits impact retinal health and how metabolic alterations can interfere with the function of not only RGCs, but also additional cell types associated with optic atrophy in FD. These findings may be applicable to other retinal diseases that are associated with mitochondrial and metabolic-mediated deficits.

## Data Availability

The data presented within this study are available upon reasonable request from the corresponding author.

## Supplementary Materials

The following supporting information can be downloaded at: https://www.mdpi.com/article/10.3390/metabo14080423/s1, Figure S1: Representative 1D ^1^H NMR spectrum of water-soluble metabolites extracted from a retina sample collected from a control mouse at P60; Figure S2: Volcano plot identifying the 9 polar metabolites whose levels were decreased in the P30 CKO retinas compared to controls; Figure S3: Representative retinal flat mount immunolabeled against anti-RBPMS at P7 and P30, indicating a significant reduction of RGCs in the Elp1 CKO FD mouse at P30 compare to control. Table S1: List of polar (i.e., water soluble) metabolites identified and quantified in mouse retinae at P7. Table S2: List of polar (i.e., water soluble) metabolites identified and quantified in mouse retinae at P60. Table S3: List of metabolites whose levels changes were found to be significant (FC > 1.5 and adjusted *p* values < 0.05) between Elp1 CKO FD mouse and control mouse retina samples as a result of a Volcano plot analysis.
