# Supplementary material for: Metabolic Deficits in the Retina of a Familial Dysautonomia Mouse Model
Source: Metabolites. 2024 Jul 31;14(8):423. doi: 10.3390/metabo14080423 (PMC11356057; doi:10.3390/metabo14080423)
Supplement: Supplementary file 1 [file metabolites-14-00423-s001.zip › metabolites-3026068-supplementary.pdf]

Supplementary Information: Costello, Shultz et al: "Metabolic Deficits in the Retina of a Familial Dysautonomia Mouse Model"

The supplementary material included herein consists of the following:

(A) Supplementary Figures

Figure S1: Representative 1D ^{1}H NMR spectrum of water-soluble metabolites extracted from a retina sample collected from an CKO at P60, recorded on a Bruker 600 MHz (^{1}H Larmor frequency) solution NMR spectrometer at Montana State University.

Figure S2: Volcano plot identifying the 9 polar metabolites whose levels were decreased in the P30 CKO retinas compared to controls.

Figure S3: Representative retinal flat mount immunolabeled against anti-RBPMS as a marker of retinal ganglion cells at two different time points, post-natal days P7 and P30.

(B) Supplementary Tables:

Table S1. Polar (i.e. water soluble) metabolites identified and quantified in mouse retinae at P7.

Table S2. Polar (i.e. water soluble) metabolites identified and quantified in mouse retinae at P60.

Table S3. List of metabolites whose levels changes were found to be significant (FC > 1.5 and adjusted *p* values < 0.05) between Elp1 CKO FD and control mouse retina samples as a result of Volcano plot analysis.

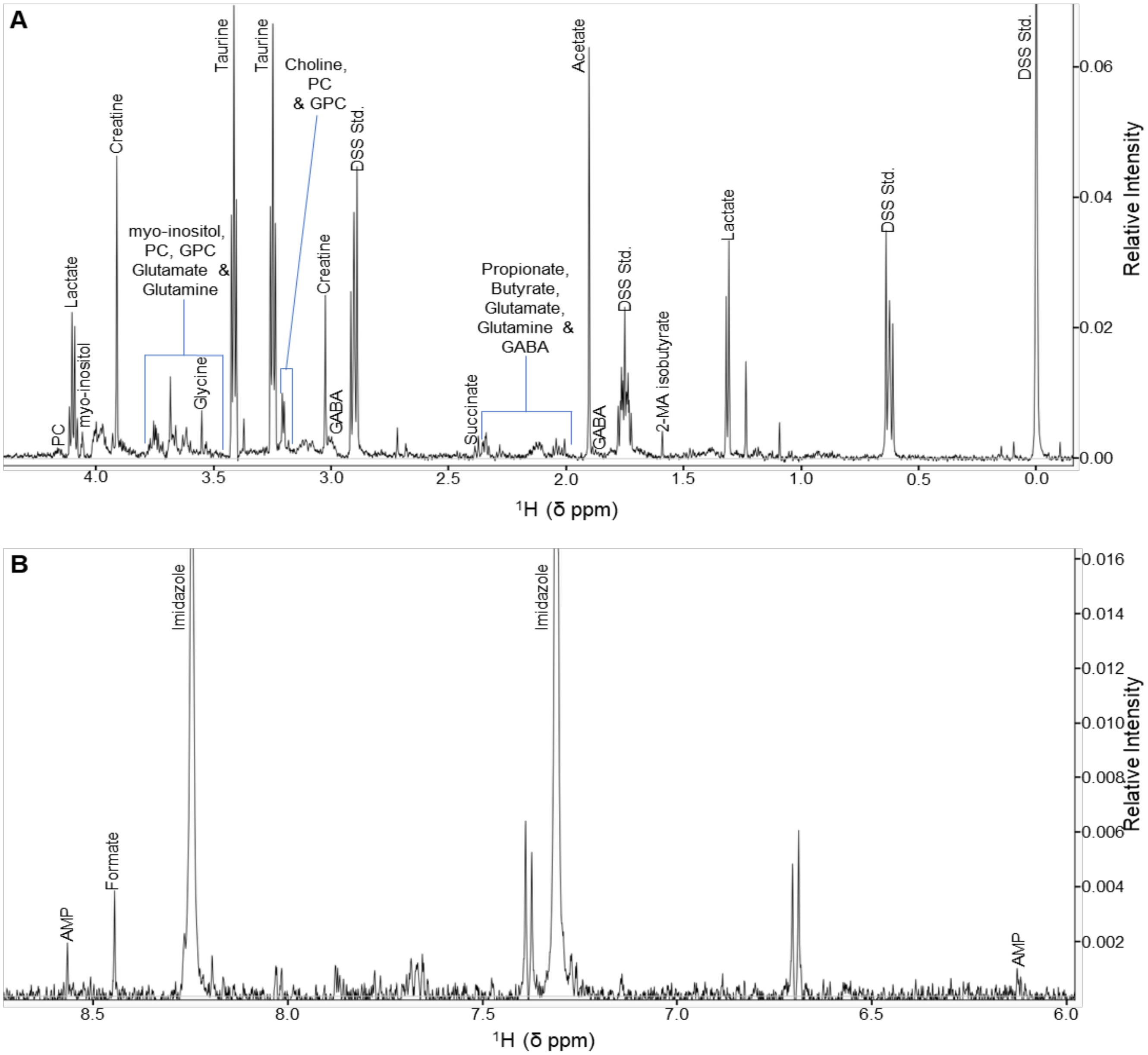

Supplementary Figure S1: Representative 1D 1H NMR spectrum of metabolite mixtures extracted from a CKO retina sample at P60, recorded on MSU's 600 MHz (14.1 Tesla) NMR spectrometer. ^{1}H NMR spectral regions corresponding to the chemical shift (δ) ranges of **(A)** 0.0 to 4.5 ppm and **(B)** 6.0 to 9 ppm are depicted. Abbreviations denote: DSS, 4,4-dimethyl-4-silapentane-1-sulfonic acid; GPC, glycerophosphocholine; PC, phosphocholine; GABA, gamma-aminobutyric acid. AMP, adenosine monophosphate; 2-MA isobutyrate, 2-methylamino isobutyrate. The x-axis denotes ^{1}H chemical shift range, and y-axis depicts a relative signal intensity scale.

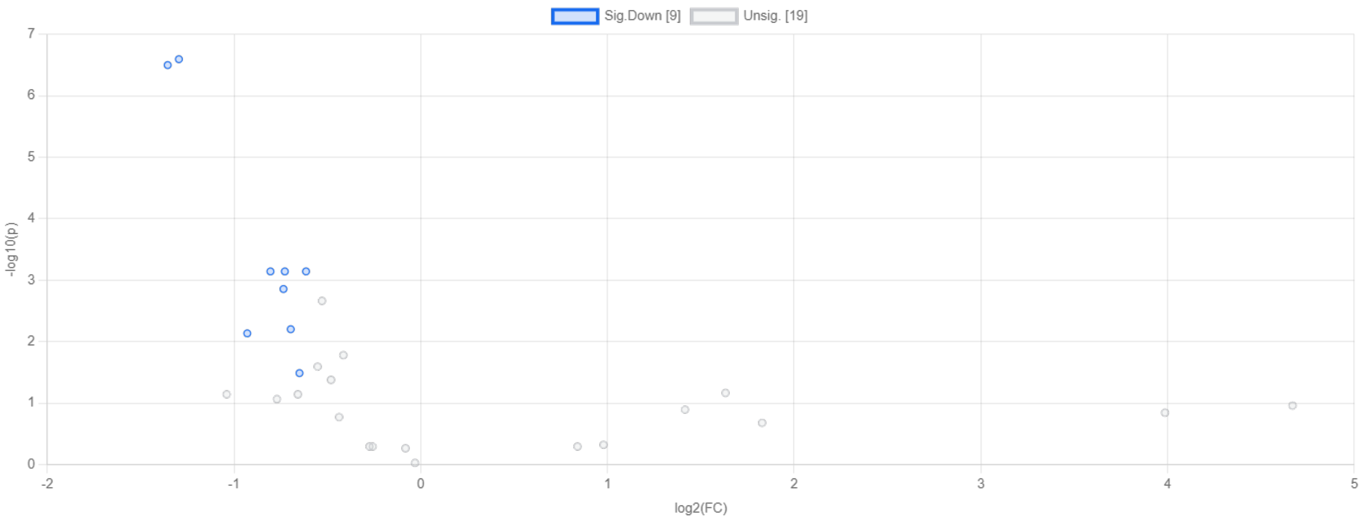

Supplementary Figure S2: Volcano plot identifying the 9 polar metabolites reported in Table S3 whose levels were decreased in the P30 CKO retinas compared to controls. The plot was generated with a false discovery rate (FDR) *p*-value threshold of 0.05 and FC threshold of 1.5. Results are plotted with y = -log10(p) and x = log2(FC). Each point represents one metabolite.

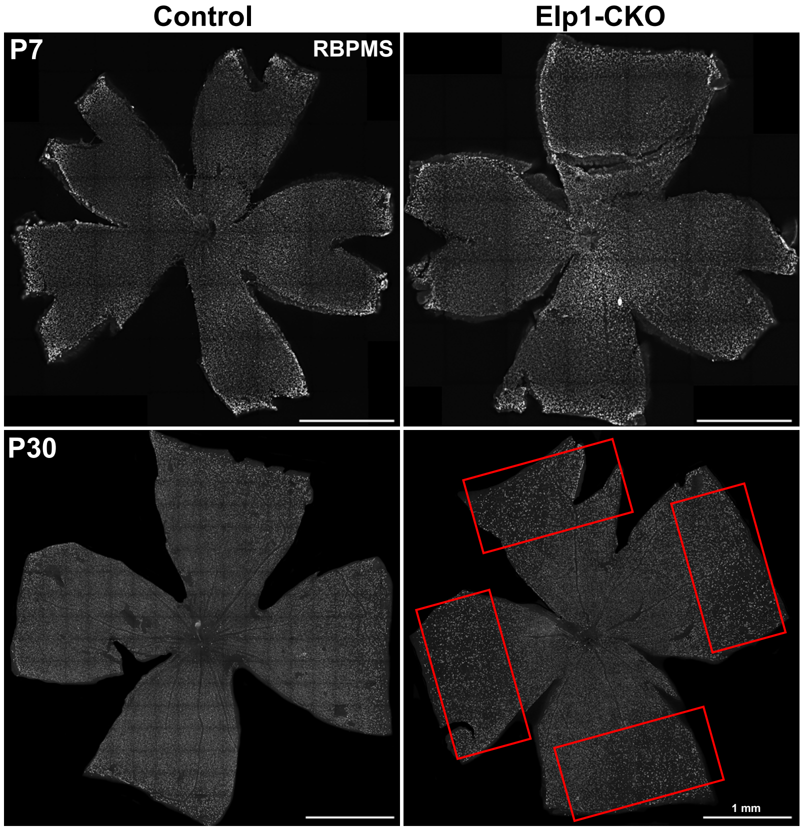

Supplementary Figure S3: Representative retinal flat mount immunolabeled against anti-RBPMS at two different time points, P7 and P30. Red boxes indicate regions of retinal ganglion cell (RGC) degeneration in the (*Pax6-cre;Elp1*$^{LoxP/LoxP}$) CKO mouse compared to littermate (*Elp1*$^{LoxP/LoxP}$) control at P30. These images demonstrate the statistically significant death of RGCs found in the (*Pax6-cre;Elp1*$^{LoxP/LoxP}$) CKO FD mouse model at P30. Scale bar, 1mm. It is also important to note that RGCs are present in far greater densities in the retina than TH+ cells.

Supplementary Table S1: Table of metabolites identified and quantified by NMR for Elp1 CKO Pax6 FD and control retinae at P7. Metabolite concentrations are reported in μM, standard deviations in μM, and fold changes are listed as the ratio of average metabolite concentrations measured in the CKO Pax6 FD mice compared to the levels measured in the control mice.

Metabolites	P7					
	CKO		Control			
	Average (μM)	SD (μM)	Average (μM)	SD (μM)	FC	Log_2 FC
Acetate	34.40	71.74	79.47	97.22	0.43	-1.21
Adenine	27.31	15.41	36.14	18.90	0.76	-0.40
AMP	6.57	6.30	8.03	8.05	0.82	-0.29
Alanine	11.14	2.22	15.37	3.48	0.72	-0.46
Aspartate	9.07	2.47	9.86	1.36	0.92	-0.12
Butyrate	8.86	20.84	25.19	30.33	0.35	-1.51
Choline	3.40	0.97	5.56	1.61	0.61	-0.71
Creatine	21.97	21.86	24.43	28.83	0.90	-0.15
Creatinine	19.69	24.35	36.60	32.47	0.54	-0.89
Glutamine	88.93	11.75	94.06	8.02	0.95	-0.08
Ethanol	7.34	5.20	9.29	6.45	0.79	-0.34
Formate	19.74	17.85	25.43	20.85	0.78	-0.37
GABA	3.31	1.88	5.56	3.14	0.60	-0.75
Glycine	29.09	6.84	30.17	4.68	0.96	-0.05
Isoleucine	1.17	0.42	1.54	0.37	0.76	-0.40
Glutamate	12.10	2.63	16.07	3.92	0.75	-0.41
Lactate	20.61	18.57	34.93	26.45	0.59	-0.76
Leucine	2.09	0.45	2.66	0.59	0.78	-0.35
Methanol	693.80	599.60	607.06	1040.74	1.14	0.19
2-MA isobutyrate	0.51	0.73	1.47	1.28	0.35	-1.52
Acetylcarnitine	0.26	0.13	0.23	0.08	1.13	0.17
Phosphocholine	17.54	3.31	21.70	2.29	0.81	-0.31
Propionate	7.59	17.25	20.74	24.72	0.37	-1.45
Succinate	1.13	0.41	1.89	1.26	0.60	-0.74
Taurine	125.87	24.76	172.86	27.44	0.73	-0.46
Valine	2.26	0.47	2.77	0.69	0.81	-0.30
myo-Inositol	5.43	1.67	7.44	1.85	0.73	-0.46
GPC	3.06	1.94	4.26	2.26	0.72	-0.48

28 metabolites were identified in retinae from 7 CKO and 7 control P7 mice. Column 1 indicates the quantified metabolite, and columns 2 and 3 report the average metabolite concentrations and standard deviations (SD) measured in the retinae from CKO and control mice, respectively. Columns 4 and 5 reports fold change (FC) and Log_2 FC of metabolite concentrations between CKO and control mice. A negative percent value indicates an average lower in CKO mice compared to control mice, and a positive percent value is higher in CKOs compared to controls. Abbreviations: AMP, adenosine monophosphate; 2-MA isobutyrate, 2-methylamino isobutyrate; GPC, glycerophosphocholine; GABA, gamma-aminobutyric acid.

Supplementary Table S2: Table of metabolites identified and quantified by NMR for Elp1 CKO Pax6 FD and control retinae at P60. Metabolite concentrations are reported in μM, standard deviations in μM, and fold changes are listed as the ratio of average metabolite concentrations measured in the CKO Pax6 FD mice compared to the levels measured in the control mice.

Metabolites	2 Months					
	CKO		Control			
	Average (μM)	SD (μM)	Average (μM)	SD (μM)	FC	Log_2 FC
Acetate	133.54	71.59	67.76	37.41	1.97	0.98
Adenine	29.18	23.39	9.41	2.05	3.10	1.63
AMP	7.67	3.94	13.08	4.77	0.59	-0.77
Alanine	2.78	0.96	3.88	0.78	0.72	-0.48
Aspartate	7.70	2.46	12.48	2.57	0.62	-0.70
Butyrate	7.27	5.90	2.73	0.46	2.67	1.42
Choline	3.41	2.34	5.35	1.92	0.64	-0.65
Creatine	25.98	15.78	53.38	12.22	0.49	-1.04
Creatinine	5.17	11.65	2.89	6.31	1.79	0.84
Glutamine	30.51	11.89	50.79	11.80	0.60	-0.74
Ethanol	0.91	0.61	1.44	0.57	0.63	-0.66
Formate	520.84	593.19	20.48	7.98	25.44	4.67
GABA	8.92	4.90	22.83	4.65	0.39	-1.36
Glycine	13.59	5.03	23.74	4.25	0.57	-0.80
Isoleucine	0.44	0.31	0.54	0.08	0.83	-0.27
Glutamate	12.48	3.17	19.10	3.10	0.65	-0.61
Lactate	56.96	15.92	77.10	28.85	0.74	-0.44
Leucine	1.12	0.17	1.19	0.26	0.95	-0.08
Methanol	14.66	38.23	0.93	0.45	15.84	3.99
2-MA isobutyrate	0.36	0.19	0.43	0.19	0.84	-0.26
Acetylcarnitine	0.29	0.12	0.55	0.16	0.53	-0.93
Phosphocholine	3.86	0.91	5.56	0.96	0.69	-0.53
Propionate	9.10	8.46	2.56	0.98	3.55	1.83
Succinate	1.36	0.27	1.99	0.61	0.68	-0.55
Taurine	271.23	50.37	361.41	70.49	0.75	-0.41
Valine	0.97	0.21	0.99	0.31	0.98	-0.03
myo-Inositol	7.18	3.69	17.61	2.61	0.41	-1.29
GPC	4.97	1.65	8.23	1.40	0.60	-0.73

28 metabolites were identified in retinae from 9 CKO and 8 control P60 mice. Column 1 indicates the quantified metabolite, and columns 2 and 3 report the average metabolite concentrations and standard deviations (SD) measured in the retinae from CKO and control mice, respectively. Columns 4 and 5 reports fold change (FC) and Log_2 FC of metabolite concentrations between CKO and control mice. A negative percent value indicates an average lower in CKO mice compared to control mice, and a positive percent value is higher in CKOs compared to controls. Abbreviations: AMP, adenosine monophosphate; 2-MA isobutyrate, 2-methylamino isobutyrate; GPC, glycerophosphocholine; GABA, gamma-aminobutyric acid.

Supplementary Table S3: List of metabolites whose fold changes are > 1.5 and adjusted FDR-corrected p-values < 0.05 found to be significant from a Volcano plot analysis of the metabolite profiles of the Elp1 CKO Pax6 FD versus Control Retinae at P60.

Name	FC	Log2(FC)	p.adjusted	-log10(p)
myo-Inositol	0.41	-1.30	2.53E-7	6.60
GABA	0.39	-1.35	3.15E-7	6.50
Glycine	0.57	-0.80	7.21E-4	3.14
GPC	0.60	-0.73	7.21E-4	3.14
Glutamate	0.65	-0.61	7.21E-4	3.14
Glutamine	0.60	-0.73	1.40E-3	2.84
Aspartate	0.62	-0.70	6.29E-3	2.20
Acetylcarnitine	0.52	-0.93	7.35E-3	2.13
Choline	0.64	-0.65	3.26E-2	1.49

Volcano plot analysis resulted in the following fold change (FC) and two-sample t-test significance. A FC threshold ≥ 1.5 and an FDR corrected p value < 0.05 were set as the criteria for significance. FC was taken as the ratio of the mean metabolite concentration for the Elp1 CKO Pax 6 mouse retinae group compared to the control mouse group prior to statistical normalization by log transformation. Abbreviations: GPC, glycerophosphocholine; GABA, gamma-aminobutyric acid.
